# Major comorbidities lead to the risk of adverse cardiovascular events in chronic obstructive pulmonary disease patients using inhaled long-acting bronchodilators: a case-control study

**DOI:** 10.1186/s12890-019-0999-z

**Published:** 2019-12-03

**Authors:** Yen-Fu Chen, Yi-Ching Cheng, Chien-Hong Chou, Chung-Yu Chen, Chong-Jen Yu

**Affiliations:** 1 Division of Pulmonary and Critical Care Medicine, Department of Internal Medicine, National Taiwan University Hospital Yunlin Branch, No.579, Sec. 2, Yunlin Rd., Douliu City, Yunlin County 640 Taiwan, Republic of China; 2 Division of Pulmonary and Critical Care Medicine, Department of Internal Medicine, National Taiwan University Hospital, and College of Medicine, National Taiwan University, Taipei, Taiwan

**Keywords:** Cardiovascular disease, Cardiovascular events, Chronic kidney disease, Chronic obstructive pulmonary disease, Comorbidity, Long-acting bronchodilator

## Abstract

**Background:**

While inhaled bronchodilators reduce symptoms and acute exacerbations of chronic obstructive pulmonary disease (COPD), their use is associated with increased cardiovascular events in some studies. This study investigates the risk of adverse events associated with the use of inhaled bronchodilators in COPD patients with multimorbidity.

**Methods:**

A case-control study was conducted between January 2015 and December 2017, and patients with spirometry-confirmed diagnosis of COPD (*N* = 1565) using inhaled long-acting bronchodilators were enrolled. Medical records were reviewed and clinical data, including age, gender, smoking status, major comorbidities, lung function stage, history of exacerbations, bronchodilator regimens, and treatment duration were analyzed. Major adverse cardiovascular events occurring during long-acting bronchodilator use were recorded.

**Results:**

The most common comorbidities were cardiovascular disease (CVD) (53.6%) and chronic kidney disease (CKD) (25.8%). We observed that CVD (odds ratio [OR], 5.77), CKD (OR, 2.02) and history of frequent exacerbations (OR, 2.37) were independent risk factors for cardiovascular events, regardless of the type of bronchodilators use. Moreover, COPD patients with both CKD and CVD had higher risk (6.32-fold) of adverse cardiovascular effects than those with neither comorbidity. Eighty-seven of 1565 (5.56%) COPD patients died during this study period. Of them, 21.8% (19/87) were cardiovascular-related and 73.6% (64/87) patients were respiratory-related mortality. Among COPD patients using long-acting bronchodilators, CKD was the only risk factor to predict cardiovascular events and cardiovascular-related mortality (OR, 4.87; 95% confidence interval [CI], 1.75–13.55].

**Conclusions:**

COPD patients had higher risk of cardiovascular events were associated with their CVD and/or CKD comorbidities and history of frequent exacerbations, rather than associated with their use of inhaled bronchodilators.

## Background

Chronic obstructive pulmonary disease (COPD) is a complex respiratory disorder characterized by chronic airflow limitations and increased inflammatory responses in the airways [1]. Comorbidities are frequent in COPD and significantly affect patient’s quality of life, exacerbation frequency, and survival [1, 2]. The most prevalent comorbidities include cardiovascular disease (hypertension, ischemic heart disease, heart failure), metabolic syndrome (diabetes mellitus, hyperlipidemia), osteoporosis and chronic kidney disease (CKD) [1, 3].

Inhaled bronchodilators, including long-acting β2-agonists (LABA) and long-acting muscarinic antagonists (LAMA), and inhaled corticosteroids (ICS) are the cornerstone therapies for COPD patients [1]. The clinical efficacy of inhaled bronchodilators has been demonstrated in clinical trials as quality of life improvement, prevention of lung function decline, and reduction of acute exacerbation frequency. However, several studies have raised concerns that inhaled bronchodilators increase the risk of cardiovascular events [4–10]. Moreover, renal impairment is also a common comorbidity in elderly COPD patients [11, 12] who may be at higher risk of adverse events due to decreased elimination and increased systemic effects of long-acting bronchodilators [13–15]. However, in large clinical trials, COPD patients with significant renal impairment or cardiac disease were usually excluded from the studies [16–18], therefore, the safety issue of COPD patient with significant renal or cardiovascular disease using long-acting bronchodilators is still being debated [9, 10, 15, 16, 19–21].

A recent analysis showed that COPD patients with CKD had high risk of pre-existing cardiovascular comorbidity [21] and they found that the safety and tolerability of dual bronchodilator is comparable to the monocomponents, irrespective of the level of renal impairment. However, the number of patients with moderate to severe renal impairment at baseline in the study remained low (less than 15%), which may not reflect the conditions in real-life practice. Moreover, the adverse effects and the cause of mortality might not be well documented among COPD patients with major comorbidities using various inhaled medications in real-life care. Here we conducted a case-control study to investigate the association of common inhaled medications, including combining different classes of bronchodilators (LAMA and LABA) and different ICS, and the development of clinically important cardiovascular events and outcomes in COPD patients with major comorbidities.

## Methods

### Study population

This study enrolled COPD patients who received inhaled long-acting bronchodilators from 2015 to 2017 at National Taiwan University Hospital (NTUH) Yunlin Branch. Eligible patients were ≥ 40 years of age, with a clinical diagnosis of COPD verified by spirometry defined as a post-bronchodilator forced expiratory volume in 1 second (FEV_1_)/forced vital capacity (FVC) ratio (FEV_1_/FVC) ≤ 0.7 and treatment with either LAMA (tiotropium, RESPIMAT®), LABA (olodaterol RESPIMAT®), LAMA/LABA (umeclidinium/vilanterol and glycopyrronium/indacaterol), ICS/LABA (fluticasone/salmeterol and budesonide/formoterol) and triple therapies (ICS/LABA with tiotropium or LAMA+LABA with budesonide). Key exclusion criteria include the patients with incomplete spirometry data, clinical diagnosis of current asthma, maintenance treatment less than 30 days of continuous use or patients who inappropriately received overlapped bronchodilators (defined as patients receiving more than two bronchodilators or wrong dual or triple combinations, for example, LAMA with LABA/LAMA combination, ICS/LABA with LABA combination, ICS/LABA with other ICS combinations or two different classes of LAMA combinations... etc.) in the same period of time.

Medical records were reviewed and clinical data, including age, gender, smoking status, comorbidities, lung function stage, history of exacerbations in the previous years, bronchodilator regimens, and treatment duration were analyzed. Major adverse cardiovascular events occurring after long-acting bronchodilator inhalation and inhaled ICS were recorded.

### Definition of major comorbidities, cardiovascular events and outcomes

Cardiovascular diseases (CVD) were coded from medical records and included hypertension, heart failure, coronary artery disease, and arrhythmia. Metabolic diseases included hyperlipidemia and diabetes mellitus (DM). The estimated creatinine clearance rate (Ccr) was calculated using the Cockcroft–Gault formula, and patients with an estimated Ccr < 60 mL/min for 3 or more months, with or without identifiable kidney damage were defined having chronic kidney disease [22]. Cardiovascular events included tachyarrhythmia, ischemic heart disease, decompensated heart failure, and cerebrovascular stroke. Causes of mortality including sepsis, respiratory-related as acute exacerbation of COPD and pneumonia, and cardiovascular-related as sudden onset of cardiac arrest, acute myocardial infarction, acute decompensated heart failure, and cerebrovascular stroke.

### Statistical analysis

Baseline characteristics of COPD patients are presented as the median with the range and percentage. One-way ANOVA was used to compare the means between patients under different long-acting bronchodilators and their combined with or without inhaled ICS treatment. Chi-square tests with Pearson values and odds ratios for categorical variables were used to investigate the risk factors of cardiovascular effects and cerebral strokes.

The association between cardiovascular events and clinical factors including basic characteristics, underlying comorbidities, and bronchodilators use was determined using conditional logistic regression for multivariate analysis. We also compared the cardiovascular risk and cerebral strokes associated with underlying comorbidities under inhaled bronchodilators use. All statistical tests were 2-sided, with statistical significance defined as *p* < 0.05. Analyses were performed using commercially available software (SPSS, version 22; IBM).

## Results

### Clinical characteristics of COPD patients

From the year 2015 to 2017, a total of 5130 patients were treated for COPD at National Taiwan University Hospital Yunlin Branch. Of them, 1561 patients lacked lung function spirometry data, and 813 patients were excluded for lung function with FEV_1_/FVC ≥ 0.7. Other exclusions include 98 patients with age < 40 years old, 278 patients receiving less than 30 continuous days of bronchodilator inhalation, and 815 patients who inappropriately receiving overlapped bronchodilators. Finally, a total of 1565 COPD patients were enrolled for further analysis.

The basic characteristics of patients with spirometry-confirmed diagnosis of COPD receiving inhaled long-acting bronchodilators are shown in Table 1. The median age was 73 years, and the majority of subjects (78.2%) were male. The major comorbidities, including hypertension (35.0%), CKD (25.8%), diabetes mellitus (18.0%) and hyperlipidemia (8.4%) were similarly distributed in each treatment cohorts. The most common inhaled therapy exposure for the COPD patients were LAMA (48.6%), followed by ICS/LABA (35.5%), LAMA/LABA (25.9%), triple therapy (13.5%) and LABA (10.3%). Regarding inhaler switching, majority of patients (*n* = 1185, 75.7%) exposed to only one inhaler, 254 (16.2%) patients exposed to two kinds of inhalers (inhaler switching once), 104 (6.6%) patients exposed to three kinds of inhalers (inhaler switching twice) and 22 (1.4%) patients exposed to 4 kinds of inhalers (inhaler switching three times). We classified the patients into different groups (LAMA, LABA, LABA/LAMA, ICS/LABA or triple therapy) according to the inhalers they were prescribed more than 30 days and recorded the events under the inhalers use. Therefore, some patients would be reclassified to different groups after inhaler switching.

**Table 1:** Characteristics of 1565 spirometry-confirmed COPD patients receiving inhaled long-acting bronchodilators

Characteristics	All	LAMA	LABA	LAMA + LABA	LABA + ICS	Triple therapy
Numbers, (%)	1565	760 (48.6)	161 (10.3)	405 (25.9)	556 (35.5)	211 (13.5)
Age (years), median (range)	73 (40–98)	73 (42–96)	75 (43–94)	73 (40–93)	71.5 (40–98)	77 (42–97)
Gender						
Male	1224 (78.2)	643 (84.6)	137 (85.1)	360 (88.9)	362 (65.1)	176 (83.4)
Female	341 (21.8)	117 (15.4)	24 (14.9)	45 (11.1)	194 (34.9)	35 (16.6)
Body mass index, median (range)	24.2 (11.0–47.5)	24.1 (11.2–46.2)	23.5 (11.2–45.5)	23.8 (12.0–44.1)	24.5 (12.0–47.5)	23.2 (12.8–44.2)
CAT score, N(%)	424 (27.1)	240 (31.6)	67 (41.6)	173 (42.7)	121 (21.8)	73 (34.6)
Mean (SD)	7.74 (6.35)	7.44 (6.39)	9.67(8.02)	9.41 (7.21)	7.06 (6.12)	8.85 (6.04)
History of exacerbations in the previous year						
0	1127 (72.0)	546 (71.8)	100 (62.1)	251 (62.0)	406 (73.0)	124 (58.8)
1	316 (20.2)	150 (19.7)	37 (23.0)	104 (25.7)	111 (20.0)	58 (27.5)
> 2	122 (7.8)	64 (8.4)	24 (14.9)	50 (12.3)	39 (7.0)	29 (13.7)
Smoking Status						
Current	266 (17.0)	137 (18.0)	30 (18.6)	81 (20.0)	80 (14.4)	37 (17.5)
Ex-smoker	530 (33.9)	284 (37.4)	71 (44.1)	186 (45.9)	143 (25.7)	90 (42.7)
Never smoker	769 (49.1)	339 (44.6)	60 (37.3)	138 (34.1)	333 (59.9)	84 (39.8)
Spirometry (FEV_1_, %)						
≥ 80	505 (32.3)	315 (41.4)	47 (29.2)	75 (18.5)	152 (27.3)	36 (17.1)
50–79	604 (38.6)	257 (33.8)	53 (32.9)	159 (39.3)	249 (44.8)	55 (26.1)
30–49	360 (23.0)	146 (19.2)	45 (28.0)	132 (32.6)	127 (22.8)	80 (37.9)
≤ 29	96 (6.1)	42 (5.5)	16 (9.9)	39 (9.6)	28 (5.0)	40 (19.0)
Underlying Comorbidities						
Metabolic disease						
Diabetes mellitus	282 (18.0)	145 (19.1)	31 (19.3)	72 (17.8)	95 (17.1)	43 (20.4)
Hyperlipidemia	131 (8.4)	77 (10.1)	11 (6.8)	41 (10.1)	51 (9.2)	12 (5.7)
Cardiovascular disease						
Hypertension	548 (35.0)	260 (34.2)	61 (37.9)	144 (35.6)	196 (35.5)	93 (44.1)
Coronary artery disease	149 (9.5)	74 (9.7)	19 (11.8)	47 (11.6)	59 (10.6)	21 (10.0)
Heart failure	93 (5.9)	46 (6.0)	12 (7.5)	25 (6.2)	34 (6.1)	15 (7.1)
Arrhythmia	50 (3.2)	24 (3.2)	7 (4.3)	12 (3.0)	18 (3.2)	8 (3.8)
Chronic kidney disease	360/1393 (25.8)	178/690 (25.8)	39/152 (25.7)	102/370 (27.6)	114/479 (23.8)	55/197 (27.9)
Malignancy						
Lung cancer	38 (2.4)	22 (2.9)	7 (4.3)	13 (3.2)	11 (2.0)	2 (0.9)
Other cancer	27 (1.7)	8 (1.1)	2 (1.2)	6 (1.5)	9 (1.6)	6 (2.8)

*LAMA* long-acting muscarinic antagonists, *LABA* long-acting beta2-agonists, *ICS* inhaled corticosteroids, *SD* Standard deviation, *FEV*_*1*_ forced expiratory volume in 1 second, *CAT* COPD Assessment Test

### Major comorbidities lead to adverse cardiovascular events

During long-acting bronchodilator inhalation treatment, patients experienced a total of 141 cardiovascular events, including 80 ischemic heart disease attacks, 39 decompensated heart failure, 5 arrhythmia, and 17 cerebral vascular strokes. The associations of clinical characteristics with cardiovascular effects (*n* = 124) and cerebral vascular strokes (*n* = 17) in COPD patients are shown in Table 2. Our data showed that male patients (odds ratio [OR], 2.76; *p* = 0.001), current or ever smoker (OR, 1.59; *p* = 0.015), higher body mass index (BMI) (≥ 27: OR, 1.81; *p* = 0.002), poor pulmonary function (FEV_1_ < 50%: OR, 1.54; *p* = 0.025), history of frequent exacerbations (exacerbation > 2: OR, 3.59; *p* < 0.001) and with underlying comorbidities, including diabetes mellitus (OR, 1.90; *p* = 0.002), hyperlipidemia (OR, 2.96; *p* < 0.001), CVD (OR, 7.76; *p* < 0.001), and CKD (OR, 2.86; *p* < 0.001) would have higher risk of adverse cardiovascular effects. The only risk factor for cerebral vascular stroke was the patients with underlying CVD (OR, 6.50, *p* < 0.001). Furthermore, COPD patients who used LAMA for long-term control had a higher risk of adverse cardiovascular effects (OR, 1.75; *p* = 0.003). In contrast, COPD patients who treated with ICS/LABA had a lower risk of adverse cardiovascular effects (OR, 0.64; *p* = 0.031). The duration of treatment was not significantly associated with cardiovascular events for any of the maintenance therapies (Table 2).

**Table 2:** The risk of cardiovascular effects and cerebral vascular strokes in COPD patients

Clinical characteristics	Cardiovascular effects (*n* = 124)		Cerebral vascular stroke (*n* = 17)	
	Odds ratio	*P* value	Odds ratio	*P* value
Male	2.76	0.001*	0.90	0.861
Age ≥ 65 y/o	1.52	0.082	2.41	0.230
Current or ever smoker	1.59	0.015*	0.52	0.197
Body mass index (BMI)				
BMI < 18.5	0.46	0.089	2.50	0.141
BMI ≥ 27	1.81	0.002*	0.60	0.582
FEV_1_ < 50%	1.54	0.025*	0.75	0.609
History of exacerbations in the previous year				
1	2.17	< 0.001**	1.42	3.31
≥ 2	3.59	< 0.001**	2.13	6.08
Major Comorbidities				
Diabetes mellitus	1.90	0.002*	0.28	0.190
Hyperlipidemia	2.96	< 0.001**	1.45	0.619
Cardiovascular disease	7.76	< 0.001**	6.50	0.001*
CKD (Ccr < 60 ml/min)	2.86	< 0.001**	1.20	0.735
Bronchodilators and treatment duration				
LAMA	1.75	0.003*	1.52	0.395
≥ 90 days	0.85	0.474	0.99	0.990
≥ 180 days	0.96	0.866	0.42	0.134
≥ 360 days	1.07	0.762	0.32	0.120
LABA	1.12	0.702	N/A	0.160
≥ 90 days	1.26	0.518	N/A	0.297
≥ 180 days	1.54	0.295	N/A	0.404
≥ 360 days	1.46	0.536	N/A	0.583
LAMA/LABA	1.46	0.057	1.57	0.373
≥ 90 days	1.01	0.974	1.96	0.200
≥ 180 days	1.07	0.788	1.27	0.711
≥ 360 days	1.12	0.727	2.48	0.144
ICS/LABA	0.64	0.031*	0.39	0.121
≥ 90 days	0.87	0.504	0.59	0.352
≥ 180 days	0.99	0.953	0.74	0.601
≥ 360 days	1.05	0.839	1.00	0.994
Triple therapy	0.95	0.844	1.38	0.613
≥ 90 days	1.02	0.951	0.59	0.607
≥ 180 days	0.99	0.983	0.71	0.735
≥ 360 days	0.79	0.588	N/A	0.297

*FEV*_*1*_ forced expiratory volume in 1 second, *CKD* chronic kidney disease, *LAMA* long-acting muscarinic antagonists, *LABA* long-acting beta2-agonists, *ICS* inhaled corticosteroids

*p < 0.05

**p < 0.001

**Fig. 1:**
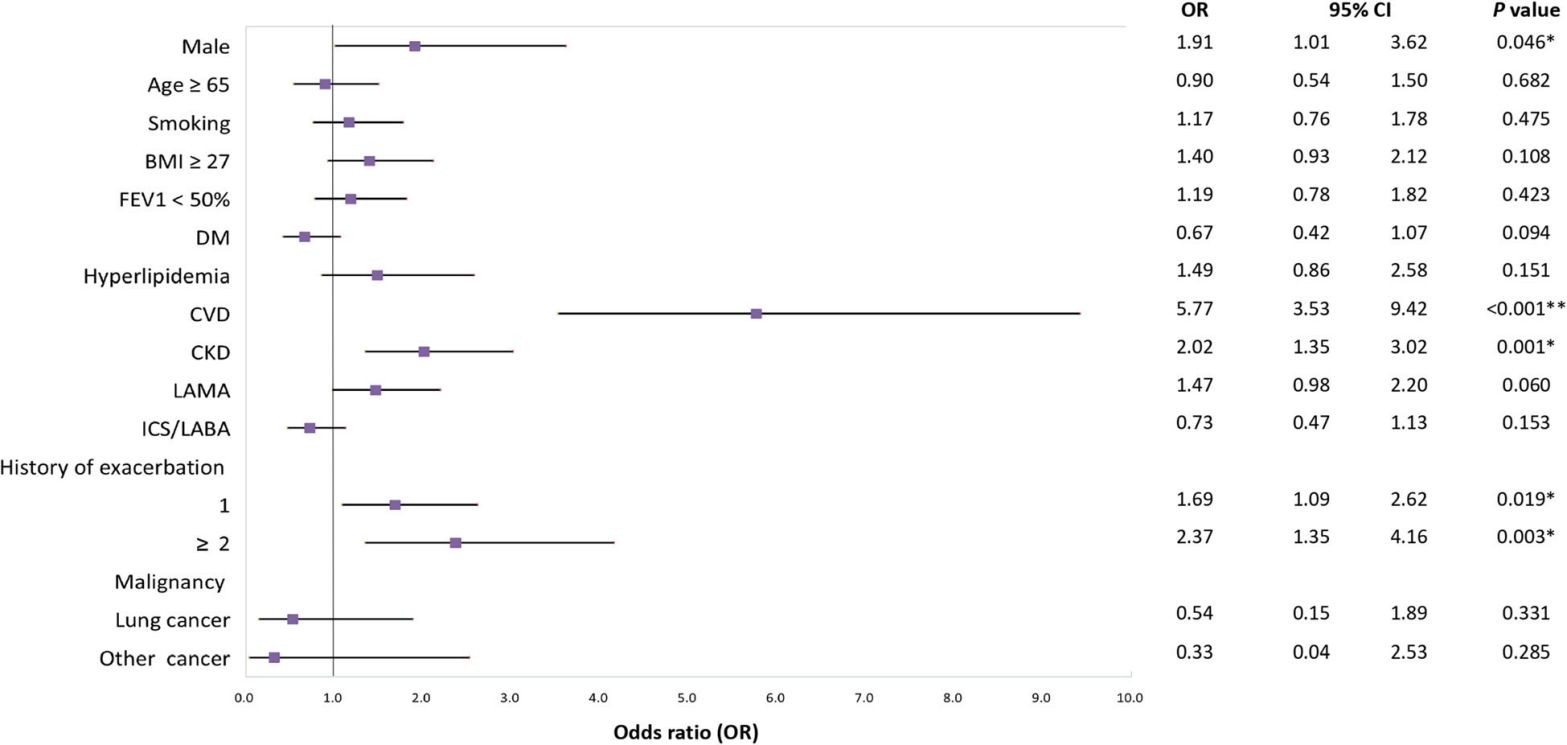
Multivariate analysis of clinical characteristics associated with adverse cardiovascular events. BMI, body mass index; FEV_1_, forced expiratory volume in 1 second; DM, diabetes mellitus; CVD, cardiovascular disease; CKD, chronic kidney disease; LAMA, long-acting muscarinic antagonists; LABA, long-acting beta2-agonists; ICS, inhaled corticosteroids; CI: confidence interval. ** *p* < 0.001. *p < 0.05

In COPD patients using bronchodilators in multivariate analysis, CVD, CKD and history of frequent exacerbations were independent risk factors for cardiovascular events (Fig. 1). The increased risk of adverse cardiovascular events with LAMA use noted in univariate analysis, notably not significant in multivariate analysis. Of the comorbidities, CVD (OR, 5.77; *p* < 0.001) and CKD (OR, 2.02; *p* = 0.001) were the major comorbidities associated with adverse cardiovascular events in COPD patients using inhaled long-acting bronchodilators. Moreover, for COPD patients with both CKD and CVD, the risk of adverse cardiovascular events increased to 6.32-fold over that of those with neither comorbidity. In the remaining COPD patients, the risk of adverse cardiovascular events increased 1.02-fold over that of those without CKD for each 1 mL/min decrease in renal creatinine clearance.

To further clarify the impact of major comorbidities, including cardiovascular disease and chronic kidney disease, as well as different inhaled therapies on the cardiovascular events in COPD patients, we compared the incidence and the risk of events between patient with or without major comorbidities stratified by five different inhaled bronchodilator combinations. In Table 3, the risk of cardiovascular effects was significantly higher among those with CVD or CKD than those without respectively, regardless of the type of bronchodilators for COPD patients.

**Table 3:** The overall incidence of cardiovascular events and stroke in COPD patients with cardiovascular disease and chronic kidney disease

	Cardiovascular events, n (%)	Odds ratio	*p*	Strokes, n (%)	Odds ratio	*p*
LAMA						
With CVD (*n* = 317)	60 (18.9)	6.23	< 0.001**	9 (2.8)	12.92	0.002*
Without (*n* = 443)	16 (3.6)			1 (0.2)		
With CKD (*n* = 178)	36 (20.2)	3.08	< 0.001**	2 (1.1)	0.72	0.673
Without (*n* = 512)	39 (7.6)			8 (1.6)		
LABA						
With CVD (*n* = 78)	13 (16.7)	16.4	0.001*	0 (0.0)		
Without (*n* = 83)	1 (1.2)			0 (0.0)		
With CKD (*n* = 39)	7 (17.9)	3.34	0.027*	0 (0.0)		
Without (*n* = 114)	7 (6.1)			0 (0.0)		
LABA/LAMA						
With CVD (*n* = 183)	33 (18.0)	5.89	< 0.001**	4 (2.2)	2.46	0.287
Without (*n* = 222)	8 (3.6)			2 (0.9)		
With CKD (*n* = 102)	23 (22.5)	4.30	< 0.001**	3 (2.9)	2.68	0.215
Without (*n* = 268)	17 (6.3)			3 (1.1)		
ICS/LABA						
With CVD (*n* = 236)	30 (12.7)	15.39	< 0.001**	3 (1.3)	N/A	0.043*
Without (*n* = 320)	3 (0.9)			0 (0.0)		
With CKD (*n* = 114)	17 (14.9)	4.09	< 0.001**	0 (0.0)	N/A	0.332
Without (*n* = 365)	15 (4.1)			3 (0.8)		
Triple therapy						
With CVD (*n* = 110)	13 (11.8)	4.38	0.015*	3 (2.7)	N/A	0.095
Without (*n* = 101)	3 (3.0)			0 (0.0)		
With CKD (*n* = 55)	8 (14.5)	2.85	0.040*	1 (1.8)	1.30	0.833
Without (*n* = 142)	8 (5.6)			2 (1.4)		

*CVD* cardiovascular disease, *CKD* chronic kidney disease, *LAMA* long-acting muscarinic antagonists, *LABA* long-acting beta2-agonists, *ICS* inhaled corticosteroids

*p < 0.05

** *p* < 0.001

### Chronic kidney disease predicts risk of mortality in COPD patients with multimorbidity using long- acting bronchodilators

Eighty-seven of 1565 COPD patients died during this study period. Among them, 21.8% patients (19 of 87) had cardiovascular-related mortality and 73.6% patients (64 of 87) had respiratory-related mortality. The overall causes of death were listed in Table 4. Multivariate analysis showed that CKD (OR, 2.45; *p* = 0.001), CVD (OR, 1.73; *p* = 0.048) and history of frequent exacerbations (OR, 4.33; *p* < 0.001), lung cancer (OR, 27.24; *p* < 0.001) were independent risk factors associated with increased mortality in COPD patients using bronchodilators. However, the risk of death decreased in COPD patients with ICS/LABA use (OR, 0.32; *p* = 0.001) (Table 5a). Of the respiratory-related mortality, history of frequent exacerbations (OR, 4.61; *p* < 0.001), lung cancer (OR, 35.79; *p* < 0.001) were the independent risk factors to increase respiratory-related mortality in COPD patients using bronchodilators (Table 5b). Of the cardiovascular-related mortality, CKD (OR, 4.87; *p* = 0.002) was the independent risk factor to increase cardiovascular-related mortality significantly in COPD patients using bronchodilators (Table 5c).

**Table 4:** Causes of death among COPD patients

Mortality causes	N (%)
Total	87
Cardiovascular-related	19
Cardiac arrest	7 (8.0)
Acute decompensated heart failure	6 (6.9)
Acute myocardial infarction	3 (3.4)
Cerebral vascular stroke	3 (3.4)
Respiratory-related	64
Pneumonia with respiratory failure	38 (43.7)
COPD acute exacerbation	26 (29.9)
Sepsis	4

**Table 5:** Multivariate analysis of clinical characteristics associated with (a) all-causes mortality, (b) respiratory-related mortality, and (c) cardiovascular-related mortality, in COPD patients

	OR	95% CI		*P* value
(a) All-causes mortality				
Male	0.79	0.37	1.67	0.532
Age > 65	1.52	0.70	3.28	0.292
Smoking	0.87	0.50	1.53	0.634
BMI > 27	0.53	0.26	1.07	0.077
FEV1 < 50%	1.66	0.96	2.88	0.070
DM	0.70	0.36	1.37	0.297
Hyperlipidemia	0.58	0.22	1.58	0.289
CVD	1.73	1.00	2.97	0.048*
CKD	2.45	1.45	4.14	0.001*
LAMA	1.00	0.59	1.70	0.994
ICS/LABA	0.32	0.17	0.63	0.001*
History of exacerbations in the previous year				
1	1.43	0.78	2.63	0.243
> 2	4.33	2.20	8.50	< 0.001**
Malignancy				
Lung cancer	27.24	12.16	61.04	< 0.001**
Other cancer	19.39	7.85	47.87	< 0.001**
(b) Respiratory-related mortality				
Male	0.83	0.34	1.98	0.667
Age > 65	1.19	0.52	2.70	0.681
Smoking	0.80	0.42	1.52	0.491
BMI > 27	0.48	0.20	1.15	0.100
FEV1 < 50%	1.82	0.97	3.41	0.060
DM	0.75	0.35	1.62	0.466
Hyperlipidemia	0.53	0.16	1.73	0.295
CVD	1.64	0.88	3.06	0.116
CKD	1.52	0.82	2.82	0.185
LAMA	1.11	0.61	2.03	0.739
ICS/LABA	0.40	0.19	0.83	0.014*
History of exacerbations in the previous year				
1	2.03	1.03	4.00	0.041*
> 2	4.61	2.14	9.93	< 0.001**
Malignancy				
Lung cancer	35.79	15.65	81.82	< 0.001**
Other cancer	11.72	4.27	32.17	< 0.001**
(c) Cardiovascular-related mortality				
Male	0.79	0.19	3.33	0.745
Age > 65	3.06	0.38	24.76	0.294
Smoking	1.11	0.39	3.14	0.849
BMI > 27	0.85	0.26	2.79	0.793
FEV_1_ < 50%	1.40	0.49	4.00	0.527
DM	0.49	0.14	1.71	0.265
Hyperlipidemia	1.14	0.23	5.70	0.869
CVD	2.99	0.98	9.10	0.053
CKD	4.87	1.75	13.55	0.002*
LAMA	0.93	0.34	2.56	0.889
ICS/LABA	0.33	0.09	1.25	0.103
History of exacerbations in the previous year				
1	0.60	0.16	2.26	0.451
> 2	2.01	0.57	7.12	0.277
Malignancy				
Lung cancer	1.43	0.17	12.34	0.742
Other cancer	10.33	2.42	44.03	0.002*

*BMI* body mass index, *FEV*_*1*_ forced expiratory volume in 1 second, *DM* diabetes mellitus, *CVD* cardiovascular disease, *CKD* chronic kidney disease, *LAMA* long-acting muscarinic antagonists, *LABA* long-acting beta2-agonists, *ICS* inhaled corticosteroids, *CI* confidence interval

** *p* < 0.001; *p < 0.05

## Discussion

Our findings demonstrated that comorbid CVD or CKD but not bronchodilator use were independent risk factors for cardiovascular events in COPD patients using long-acting inhaled bronchodilators. In COPD patients with CKD, the risk of adverse cardiovascular events increased 1.02-fold over that of patients with COPD alone for each 1 mL/min decrease in renal creatinine clearance. In COPD patients with both CKD and CVD, this risk enhanced to 6.32-fold over that of those with neither comorbidity, regardless of the type of inhaled long-acting bronchodilator used. Furthermore, we found that CKD was the only independent risk factor to predict cardiovascular-related mortality in COPD patients. This is the first study to raise the possibility that the increased risk of adverse cardiovascular events among COPD patients using inhaled bronchodilators results from CVD and/or CKD as their major comorbidities and history of frequent exacerbations, rather than from the use of inhaled medications themselves.

We observed that COPD patients who used LAMA had a higher risk for cardiovascular events in univariate analysis that was not statistically significant in multivariate analysis. In contrast, prior nested case-control studies by Wang et al. [9] and Gershon et al. [10] found that COPD patients newly prescribed LAMAs or LABAs were at higher risk of cardiovascular events, irrespective of prior CVD status. This discrepancy might attribute that their potential misclassification of patients with COPD by ICD codes in the National Health Insurance Research Database (without lung function confirmation) and the majority COPD patients in the cohort did not receive standard treatment (less than 15% of the patients with COPD who had cardiovascular events had been treated with long-acting bronchodilators, and the most of them used oral theophylline, beta-agonists, and an oral corticosteroid as the initial treatment) [9], which further increased cardiovascular side effects [23, 24].

Many observational studies and meta-analyses have reported that increase cardiovascular risk in patients with COPD was associated with their use of long-acting bronchodilators [5, 19, 20, 25, 26], nevertheless, the randomized controlled trials failed to show an increased risk [16, 27, 28] and a recent study even demonstrated that a LAMA/LABA combination improves cardiac function in COPD patients with lung hyperinflation [29]. This discrepancy may be partially attributable to the fact that most clinical trails have excluded patients with severe or multiple comorbidities. One population-based study indicated that the risk of cardiovascular events after initiation of long-acting bronchodilators is 3.5-fold higher in patients with baseline CVD who are taking CVD medications [30]. Similarly, we also observed that COPD patients with baseline CVD had higher risk of cardiovascular effects than those without, regardless of treatment regimens for COPD. Further multivariate analysis demonstrated that patients with baseline CVD predicted the risk of adverse cardiovascular events. Although inhaled long-acting bronchodilators are recommended as maintenance therapy for stable COPD patients [1], clinicians should be cautious when prescribing these medications to patients with preexisting cardiovascular disease.

Recent studies showed that CKD not only has a significantly higher prevalence in COPD patients than in healthy controls [31–33], but also an important risk factor for CVD [34], which may carry high risk of cardiovascular complications [35]. The finding is of concern because LABA and LAMA are both excreted in the urine, and the systemic exposure of LABA and LAMA would be higher in COPD patients with renal impairment than those with normal renal function [14, 36]. Thus, COPD patients with CKD receiving long-acting bronchodilators might lead to severe adverse cardiovascular effects after long-term exposure [15] and even lead to significantly higher risk of death compared to those without CKD [37–39]. Our findings documented that COPD patients comorbid CKD not only have higher risk of cardiovascular events and also predict the risk of cardiovascular-related mortality. However, this data highlights the need for further prospective studies to investigate the underlying mechanisms and potential interventions to improve outcomes in this population.

Comparative studies have failed to show a significant difference in the risk of mortality and serious adverse events between LAMA, LABA, and ICS/LABA [40, 41]. Nevertheless, a systematic review and meta-analysis demonstrated that LAMA use was associated with higher risk of overall and cardiovascular death compared with other inhaled medications and ICS/LABA use was associated with the lowest risk of overall death among all treatments [42]. Differently, our reports showed that COPD patients with CKD, CVD, history of frequent exacerbations and underlying malignancies were associated with high risk of all-cause mortality, and those treated with ICS/LABA were associated with better outcome. These results imply that clinicians should not only foucs on the selection of inhaled bronchodilators for COPD, but also target the extra-pulmonary comorbidities as treatable traits to improve outcomes in real-world practice [43].

Our study has several limitations. First, our study population was extracted from only one medical center database, so the results may not be applicable to patients examined at other clinics. Second, not all the prescriptions of inhaled bronchodilators were regulated by guidelines and the data is based on retrospective chart review. Thus, the COPD treatment may not be standardized and the adherence rate of inhalers could not be assessed. Third, the observed association between cardiovascular events in patients with preexisting cardiovascular conditions and the initiation of inhaled bronchodilators does not imply cause and effect. Fourth, we did not include COPD patients with CVD who are not taking inhaled COPD therapies for further clarifying the effects of inhaled bronchodilators on patients with CVD, which is attributed to very few diagnosed COPD patients without receiving any standard inhaled long-acting bronchodilators, even they with CV comorbidity. Fifth, our cohort enrolled relatively higher percentage of nonsmoker COPD patients (49.1%), which could be explained by enrollment of patients with asthma and COPD overlapped (ACO) and our patients had long-term exposure to highest level of fine particulate matter (PM_2.5_) in Taiwan [44]. Sixth, nearly one-fourth of patients (*n* = 380, 24.3%) had inhaler switching during the follow-up period and were reclassified to different groups after inhaler switch. However, in the real-world data with retrospective nature, we could not avoid the impact of carryover effects without washout periods. Finally, we did not investigate the medications used to treat underlying comorbidities such as DM, hypertension, or hyperlipidemia, or oral drugs for COPD patients.

## Conclusions

The risk of cardiovascular events was associated with COPD patients with preexisting CKD or CVD, and history of frequent exacerbations rather than associated with the use of inhaled bronchodilators. Clinicians should closely monitor COPD patients with preexisting CKD and CVD for adverse cardiovascular events while using inhaled long-acting bronchodilators.

## Data Availability

The datasets used and/or analysed during the current study are available from the corresponding author on reasonable request.

## Abbreviations

ACO: Asthma and COPD overlapped
BMI: Body mass index
Ccr: Creatinine clearance rate
CKD: Chronic kidney disease
COPD: Chronic obstructive pulmonary disease
CVD: Cardiovascular diseases
DM: Diabetes mellitus
FEV1: Forced expiratory volume in one second
FVC: Forced vital capacity
ICS: Inhaled corticosteroids
LABA: Long-acting β2-agonists
LAMA: Long-acting muscarinic antagonists
OR: Odds ratio
PM: Particulate matter
